# Population structure and history of *Mycobacterium bovis* European 3 clonal complex reveal transmission across ecological corridors of unrecognized importance in Portugal

**DOI:** 10.1128/spectrum.03829-23

**Published:** 2024-05-21

**Authors:** André C. Pereira, José Lourenço, Gonçalo Themudo, Ana Botelho, Mónica V. Cunha

**Affiliations:** 1 Centre for Ecology, Evolution and Environmental Changes (cE3c) & CHANGE—Global Change and Sustainability Institute, Faculdade de Ciências, Universidade de Lisboa, Lisbon, Portugal; 2 Biosystems & Integrative Sciences Institute (BioISI), Faculdade de Ciências, Universidade de Lisboa, Lisbon, Portugal; 3 INIAV, I.P.—National Institute for Agrarian and Veterinary Research, Oeiras, Portugal; LSU Health Shreveport, Shreveport, Louisiana, USA

**Keywords:** *Mycobacterium bovis*, animal tuberculosis, whole-genome sequencing, phylodynamics, ecological modeling, transmission dynamics

## Abstract

*Mycobacterium bovis* causes animal tuberculosis in livestock and wildlife, with an impact on animal health and production, wildlife management, and public health. In this work, we sampled a multi-host tuberculosis community from the official hotspot risk area of Portugal over 16 years, generating the largest available data set in the country. Using phylogenetic and ecological modeling, we aimed to reconstruct the history of circulating lineages across the livestock-wildlife interface to inform intervention and the implementation of genomic surveillance within the official eradication plan. We find evidence for the co-circulation of *M. bovis* European 1 (Eu1), Eu2, and Eu3 clonal complexes, with Eu3 providing sufficient temporal signal for further phylogenetic investigation. The Eu3 most recent common ancestor (bovine) was dated in the 1990s, subsequently transitioning to wildlife (red deer and wild boar). Isolate clustering based on sample metadata was used to inform phylogenetic inference, unravelng frequent transmission between two clusters that represent an ecological corridor of previously unrecognized importance in Portugal. The latter was associated with transmission at the livestock-wildlife interface toward locations with higher temperature and precipitation, lower agriculture and road density, and lower host densities. This is the first analysis of *M. bovis* Eu3 complex in Iberia, shedding light on background ecological factors underlying long-term transmission and informing where efforts could be focused within the larger hotspot risk area of Portugal.

**IMPORTANCE:**

Efforts to strengthen surveillance and control of animal tuberculosis (TB) are ongoing worlwide. Here, we developed an eco-phylodynamic framework based on discrete phylogenetic approaches informed by *M. bovis* whole-genome sequence data representing a multi-host transmission system at the livestock-wildlife interface, within a rich ecological landscape in Portugal, to understand transmission processes and translate this knowledge into disease management benefits. We find evidence for the co-circulation of several *M. bovis* clades, with frequent transmission of the Eu3 lineage among cattle and wildlife populations. Most transition events between different ecological settings took place toward host, climate and land use gradients, underscoring animal TB expansion and a potential corridor of unrecognized importance for *M. bovis* maintenance. Results stress that animal TB is an established wildlife disease without ecological barriers, showing that control measures in place are insufficient to prevent long-distance transmission and spillover across multi-host communities, demanding new interventions targeting livestock-wildlife interactions.

## INTRODUCTION

*Mycobacterium bovis* is the causative agent of animal tuberculosis (TB) in livestock and wildlife, retaining zoonotic potential (1). The main affected livestock species worldwide is *Bos taurus* (bovine), and in the Iberian Peninsula, the main affected wildlife species are *Sus scrofa* (wild boar) and *Cervus elaphus* (red deer) (2–4). The livestock-wildlife interface is considered of key importance in the dissemination of *M. bovis* by both direct and indirect transmission routes, which are highly dependent on husbandry systems and host activity aggregation points (5–10).

In Portugal, there is a national bovine TB program aiming control towards eradication (11). It is based on three pillars: (i) detection and compulsory slaughter of animal reactors to the single intradermal comparative cervical tuberculin test, (ii) routine surveillance of carcasses at slaughterhouses, and (iii) pre-movement testing (11). The official wildlife surveillance program targets a hotspot risk area related to the synanthropy of big game species due to natural conditions and artificial management, in east-central and eastern-south mainland Portugal (5, 6, 12–14). In this area, TB prevalence in cattle (herd level) was 0.5%–1.24% in the 2007–2017 period (11); in wild boar, it ranged from 15.9% down to 5.6% in the 2008–2016 period; and, in red deer, from 10.3% to 5.0% in the 2008–2016 period (5, 15). Animal TB occurrence in the hotspot area could stem from several factors, including the extensive management practices involved in bovine husbandry; wildlife community composition and abundance, and their susceptibility to TB; and the overpopulation of large game species, such as red deer and wild boar, which are managed artificially for hunting purposes and are widely dispersed throughout the area (16). The combinatorial effect of all these factors leads to animal aggregation and enables both direct and indirect interaction of bovine with wild animals in communal pastures.

*M. bovis* epidemiological surveillance in Portugal has mostly been based on classic molecular characterization, including spoligotyping and MIRU-VNTR (6, 12, 15–18). Efforts remain focused on the three main reservoirs of TB within the hotspot risk area (19–21). Classic molecular approaches analyze only <1% of the genome and are not sufficiently discriminatory to accurately assess transmission chains. Outputs are particularly compromised by homoplasy and are insufficient to gain insights into the roles exerted by different species in the multi-host system (22, 23). Whole-genome sequencing (WGS) approaches can overcome these limitations, using single-nucleotide polymorphisms (SNPs) as reliable phylogenomic markers, identifying genetic population structures while linking to co-variables of interest, thus unraveling transmission drivers and routes (19, 24–32). Global studies have identified five main clonal complexes, with three predominant in Europe: Eu1 is globally distributed, with an origin associated with the British Isles (33); Eu2 (lineage La1.7.1) is predominant in the Iberian Peninsula (33); Eu3 was recently shown to be predominant in Western Europe and East-Africa (34).

In recent, first-of-a-kind pilot studies applying WGS, we have identified transmission between livestock (bovine) and wild ungulates (red deer, wild boar) in the Castelo Branco and Portalegre districts within the hotspot risk area of mainland Portugal (16, 19). Following this first study, a second one substantially expanded the number of existing *M. bovis* full genomes and enabled phylodynamic modeling analyses of the major clade of the European 2 clonal complex population present in Portugal (35).

In this study, under a new sampling and WGS effort over 16 years (2002–2018), we analyze 170 *M*. *bovis* full genomes within the hotspot risk area in mainland Portugal. Using phylogenetic and ecological modeling approaches informed by WGS and sample metadata, we recover and describe local transmission chains, provide robust estimations of several evolutionary parameters, and uncover an ecological corridor of previously unrecognized importance in mainland Portugal.

## RESULTS

### Population structure

**Fig 1:**
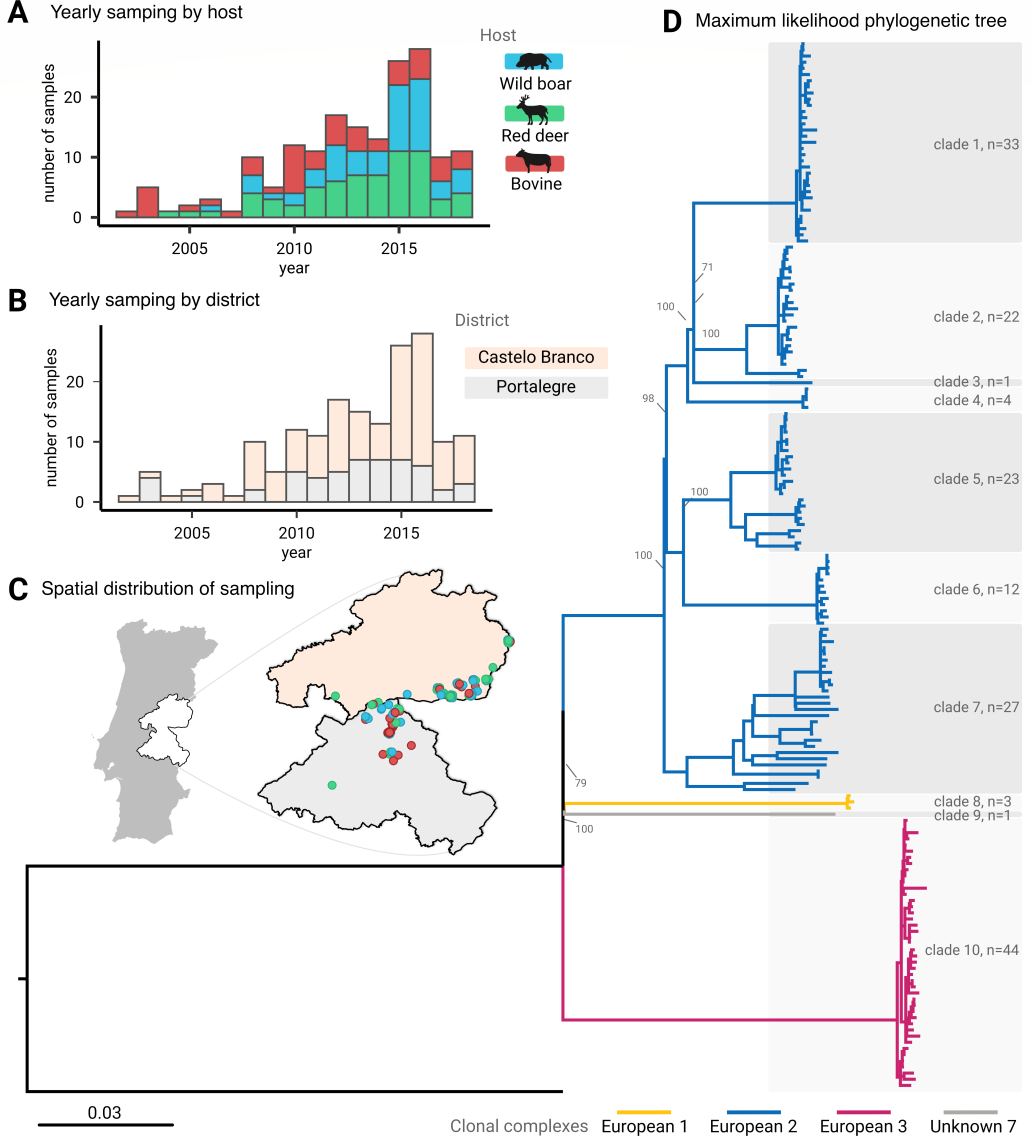
*M. bovis* (*n* = 170) sampling according to host and district and maximum likelihood phylogenetic tree. Total number of samples per year in the data set according to (**A**) host (wild boar in blue, red deer in green, bovine in red), (**B**) district (Castelo Branco in orange, Portalegre in gray), and (**C**) location. (**D**) Maximum likelihood SNP-based phylogenetic tree showing clades 1 to 10 (with sampling sizes), and color-coded major clades corresponding to clonal complexes: European 1 (yellow, including clade 8), European 2 (blue, including clades 1–7), European 3 (magenta, including clade 10), and Unknown 7 (gray, including clade 9).

The data set used in this study reflects the local surveillance history of TB, which is characterized by low sampling before 2006 due to insufficient surveillance (Fig. 1A), an increase in wildlife isolates after 2011 due to the creation of the official hotspot risk area related to wildlife game species (Fig. 1A), and by an overall higher number of isolates recovered from Castelo Branco district due to a higher hunting activity in that area (Fig. 1B and C). The phylogenetic distribution of SNPs grouped isolates into clades 1 to 10 (Fig. 1D). Clades 1 to 7 were related to Eu2, clade 8 to Eu1, clade 9 to Unk7 (unknown clonal complex 7, also known as lineage 1.8.2), and clade 10 to Eu3 (Fig. 1D; Table S2). All major clades included strains from the three host species and the Castelo Branco and Portalegre districts, suggesting mixing in transmission sources and routes.

### Transmission mapping

**Fig 2:**
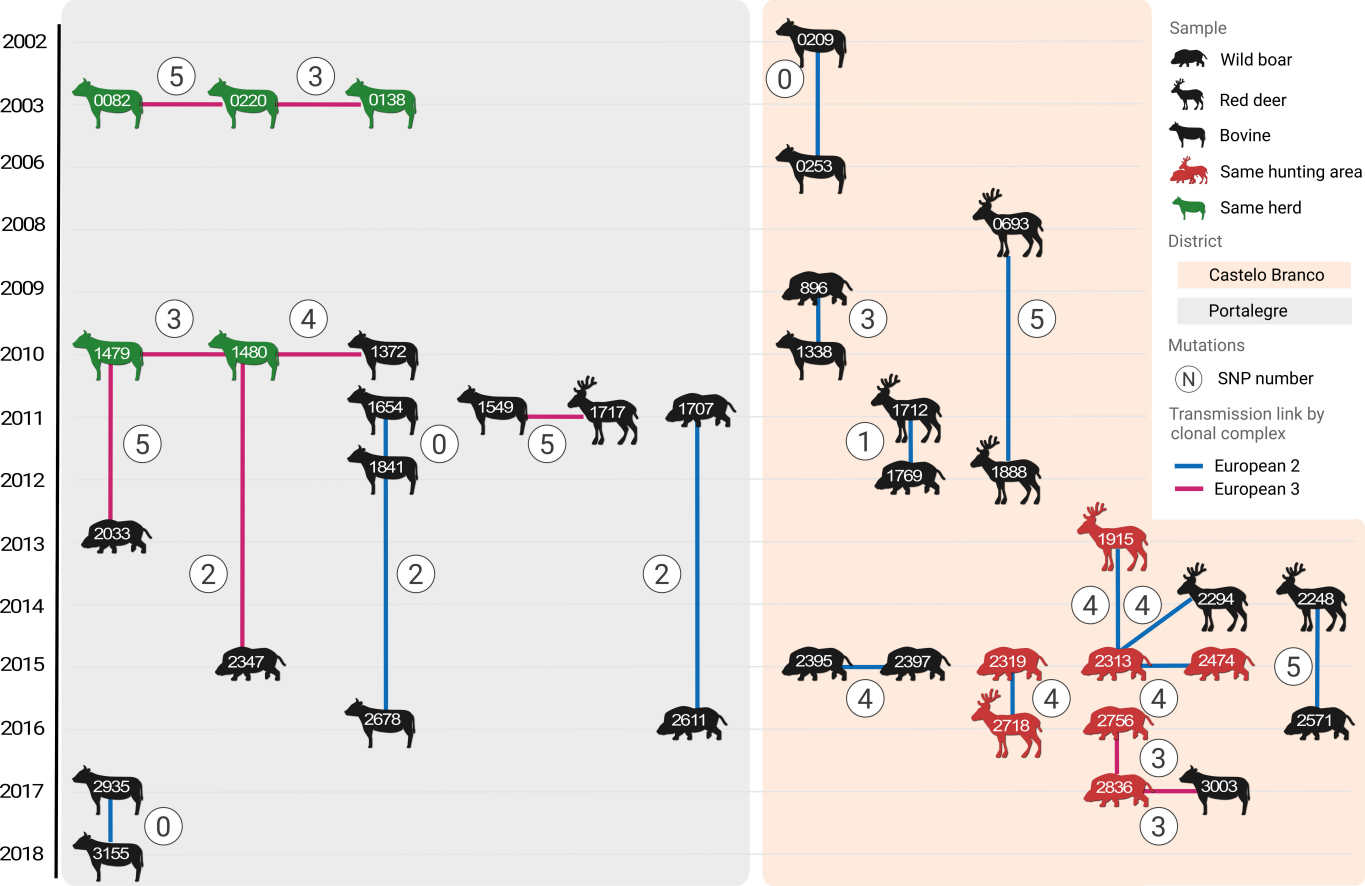
Inferred recent *M. bovis* transmission network. Diagram of the recovered transmission events according to host species (animal symbols), individuals (ID number), location (background shading), time (scale on the left), and SNP difference (circles with numbers). Lines mark transmission links between individuals according to clonal complex (line color, blue for Eu2, and pink for Eu3). Individuals within the same hunting area are in red (wildlife) and those within the same herd are in green (bovine).

Local transmission networks based on the SNP alignment and available metadata identified several transmission chains (Fig. 2). Fifteen chains were identified with 2 to 5 isolates per chain (25 events). In Castelo Branco district, there were nine transmission chains, with two at the wildlife-livestock interface, one in livestock, six in wildlife including three within the same hunting area. In Portalegre district, there were five transmission chains, with two at the wildlife-livestock interface, two in livestock involving animals from within the same herd, and one exclusive to wildlife. No inter-district transmission events were found. Only 3 in 25 inferred individual transmission events had zero SNP differences and all occurred within the same host species (bovine). Nevertheless, strains with less than 6 SNP differences were also detected within a time interval of 6–11 years even within the same hunting area.

### Phylogenomics

Root-to-tip and date-randomization analyses confirmed that Clade 10 (Eu3, Fig. 1D) presented sufficient temporal signal for further phylogenomic analyses (Fig. S2). The clade had 44 isolates (25% of isolates) from bovine (*n* = 13), red deer (*n* = 16), and wild boar (*n* = 15), from Castelo Branco (*n* = 18) and Portalegre (*n* = 26), sampled in 2003 and 2010–2018. TIM2 with invariant sites and a four categories discrete gamma-distribution (TIM2 + I + G) was the best-fitting nucleotide substitution model (Table S4).

**Fig 3:**
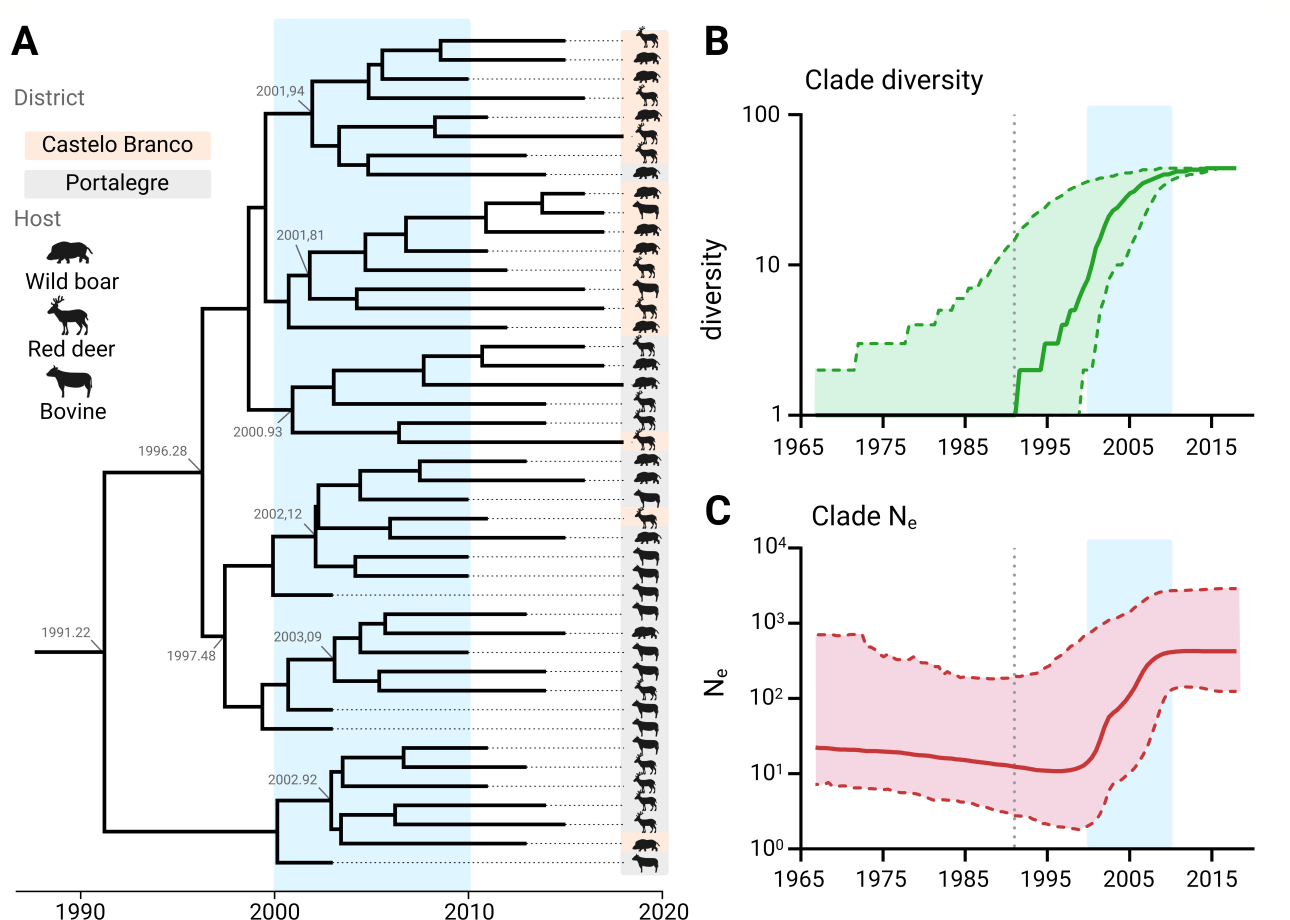
European 3 clonal complex ancestry, diversification, and effective population size. (**A**) Time-calibrated maximum clade credibility tree. Sampled individuals are presented by corresponding animal symbols and locations by shaded colors (orange for Castelo Branco, gray for Portalegre) on the right side. Numbers in gray along the tree show the branching timings of subclades. (**B**) Lineage-through-time analysis showing clade diversity in time (green), where the median is represented by a full line and the 95% confidence interval (CI) by a shaded area. (**C**) Bayesian skyline reconstruction shows effective population size in time (red), where the median is represented by a full line and the 95% CI by a shaded area. In both (**B,C**) panels, the vertical dotted line marks the clade’s tMRCA (median), and in all panels, the blue shaded area highlights a period of particular growth.

A time-based phylogeny indicated the relaxed exponential clock with a Bayesian Skyline population as the best-fitting model (Table S5). The mean clock rate of Eu3 was estimated to be 2.2 × 10^−4^ [95% highest probability density, HPD, (8.9 × 10^−5^–3.6 × 10^−4^)] substitutions per site per year, corresponding to a mean evolutionary rate of 0.2 [95% HPD (0.1–0.4)] substitutions per genome per year. The median tMRCA was estimated 28 years ago [95% HPD (16, 18–53)], corresponding to 1991 [95% HPD (1965–2001)] (Fig. 3A). A lineage through time analysis (Fig. 3B) revealed that after the tMRCA, an initial increase in strain diversity occurred, with particular growth around the turn of the century and eventual plateauing around 2010. A coalescent Bayesian Skyline analysis estimating the effective population size (*N*_e_) through time, further supported these temporal observations (Fig. 3C).

### Ancestral state host reconstruction

**Fig 4:**
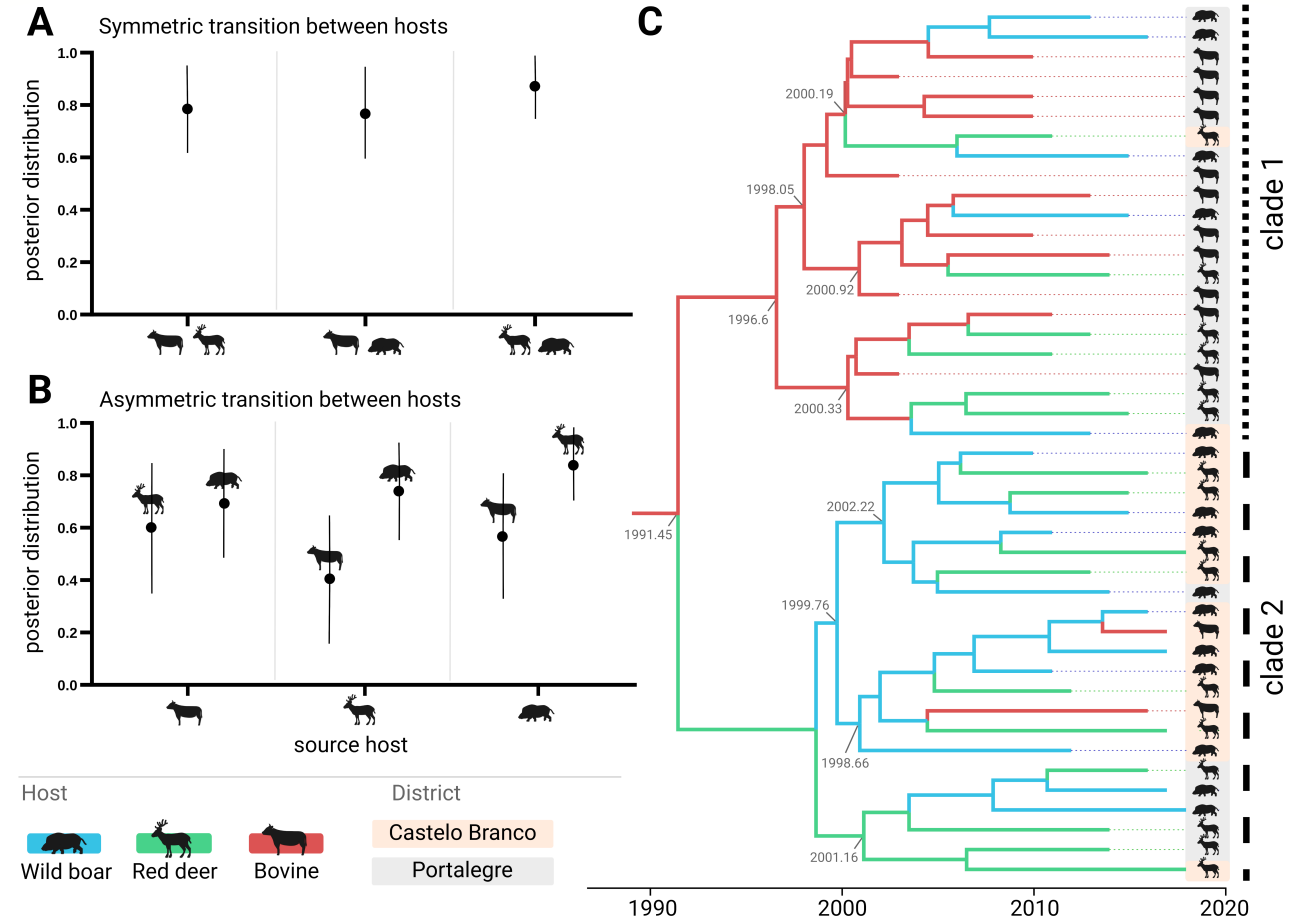
European 3 clonal complex ancestral state host reconstruction. (**A**) Host state posterior probabilities under a model of symmetric host species transitions, with source/sink hosts on the *x*-axis. (**B**) Host state posterior probabilities under a model of asymmetric host species transitions, with source host on the *x*-axis and sink host on the data points. (**C**) Maximum credibility tree estimated under a model of symmetric host species transitions. The two major clades 1 and 2 are highlighted on the right. Numbers in gray along the tree show the branching timings of subclades. In panels (**A–C**), host species are represented by animal symbols as well as colors (wild boar in blue, red deer in green, bovine in red), while location is represented by shaded background (orange for Castelo Branco and gray for Portalegre).

We estimated the internal node probability of association with each host species. The symmetric model showed the best fitting (Table S6; Fig. S3). A slightly stronger signal was inferred for host transitions between both wildlife hosts (posterior probability, PP = 0.87) compared to transitions between livestock and wildlife (bovine-red deer, PP = 0.79; bovine-wild boar, PP = 0.77) (Fig. 4A). The asymmetric model, while less supported, also estimated reasonably high transitions (PP > 0.5) between pairs of hosts, except for transitions between red deer and bovine (Fig. 4B). The MRCA was associated with bovine, subsequently bifurcating into two subclades (clades 1, 2) (Fig. 4C). Clade 1 was characterized by persistence in bovine and host transition events towards wildlife species, mostly within Portalegre, and clade 2 was characterized by persistence in wildlife, with later host transitions back to bovine, mostly within Castelo Branco.

### Phylogeography using spatial data

We next estimated the internal node probability relating to the sample’s geographic location. When comparing all phylogeographic models, using districts as grouping levels, applying a symmetric coalescent model was the most supported approach (Table S6). The probability of transitions between both districts was close to 1.00 (Fig. S3). Considering the less supported asymmetric coalescent model (Fig. S4), high probabilities of transition between districts, in both directions, were also estimated (Castelo Branco—Portalegre, PP = 0.97, Bayes Factor, BF = 1,630.91; Portalegre—Castelo Branco, PP = 1.00, BF = 7.00) (Fig. S3).

Furthermore, coalescent models using municipalities as grouping levels showed relatively low transition probabilities, in both symmetric and asymmetric models, with only five transitions showing relatively high probabilities: in the symmetric model, only within districts, transition showed high probabilities (Castelo Branco—Idanha-a-Nova, PP = 1.00, BF = 4.24; Castelo de Vide—Nisa, PP = 0.63, BF < 3.00; Castelo de Vide—Portalegre, PP = 0.62, BF < 3.00; Nisa—Portalegre, PP = 0.64, BF < 3.00) (Fig. S5); in the asymmetric model, only one transition showed high probability and it was between districts (Idanha-a-Nova—Avis, PP = 0.90, BF = 3.78) (Fig. S5). Further information is included in Text S1.

**Fig 5:**
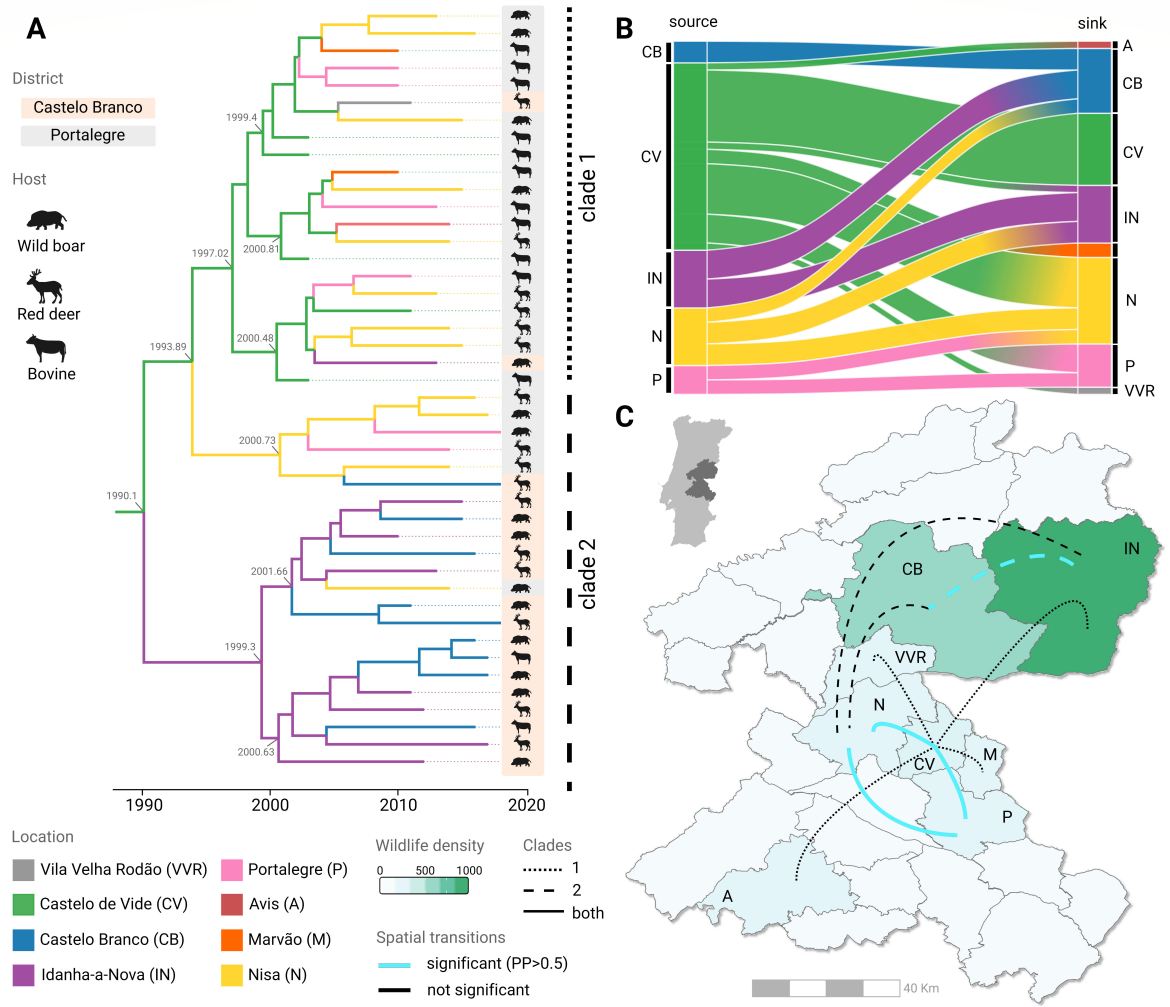
Phylogeography of the European 3 clonal complex using spatial boundaries and coordinates. (**A**) Maximum credibility tree estimated using a model of asymmetric municipality transitions. The two major clades 1 and 2 are highlighted on the right. Numbers in gray along the tree show the branching timings of subclades. Animal symbols are used to represent species and the shaded backgrounds highlight the district of sampling (orange for Castelo Branco, gray for Portalegre). (**B**) Alluvial plot denoting both intra- and inter-municipality transitions (recovered from inferences on internal nodes). In panels (**A and B**), colors are used to represent municipalities. Note that the names Portalegre and Castelo Branco are used both for districts and the respective main municipalities of those districts. (**C**) Spatial representation of inter-municipality transitions for clade 1 (dotted lines), clade 2 (shaded line), or both (full lines) on the background of wildlife density (green color scale). Transitions with high posterior probability (PP) (>0.5) are colored in light blue.

The MRCA was associated with Portalegre district (municipality Castelo de Vide) with subsequent geographic events towards Castelo Branco district (Fig. 5A). Similar to the inferred timings of major growth in diversity and effective population size (Fig. 3BC), a boost in inter-municipality transitions occurred after the turn of the 21st century. Estimated transitions between municipalities were recovered from the tree internal nodes, showing five municipalities dominating spatial transitions in time, namely Nisa (Portalegre district), Castelo de Vide (Portalegre), Portalegre (Portalegre), Idanha-a-Nova (Castelo Branco), and Castelo Branco (Castelo Branco) (Fig. 5B). Notably, these five municipalities have higher wildlife animal density (Fig. 5C). Castelo de Vide in the district of Portalegre was the municipality with most transitions, both intra- and inter-municipality.

### Phylogeography of *M. bovis* using ecological clustering data

Finally, using metadata related to the location of all available *M. bovis* isolates, we explored the underlying ecological landscape. For this, we estimated the internal node probability relating to the sample ecological cluster defined over clustering analyses including 12 ecological variables (Multiple Factor Analysis—MFA, Text S2). When comparing the tested phylogeographic models that either assumed the probability of four or five ecological clusters, the existence of four ecological clusters (I–IV) was the most supported approach (independently of the symmetry assumption used) (Table S6).

**Fig 6:**
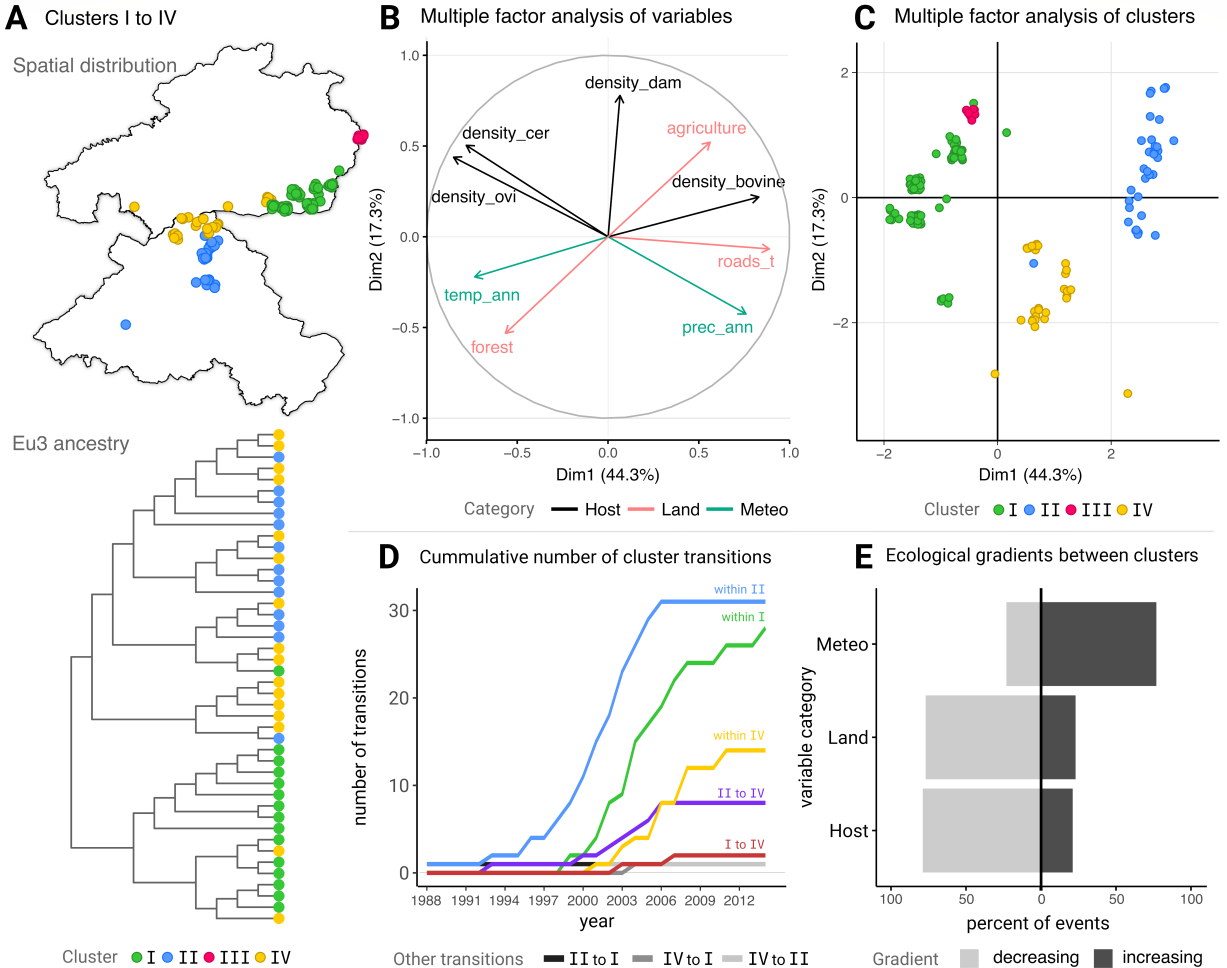
*M. bovis* ecological clustering and European 3 clonal complex cluster transitions. (**A**) Distribution of ecological clusters (I, II, III, and IV, in different colors) according to all available isolates in space (top) and for Eu3 isolates in ancestry (bottom) where each colored point is an *M. bovis* isolate. (**B**) Multiple factor analysis (MFA) presenting dimensions 1 and 2. Shown are the top nine variables that most contributed to dimensions 1 and 2. Variables and their axes are colored according to the variable category they belong to [black for those related to Host, pink when related to Land, and marine when related to Meteorology (Meteo)]. For the definition of variable categories, as for visual output including all variables, see Text S2. (**C**) Mapping of each *M. bovis* isolates in MFA dimensions 1 and 2 space, colored according to the ecological cluster. (**D**) Cumulative number of intra- and inter-cluster inferred transitions with time. (**E**) Inferred between cluster transitions are summarized depending on whether the mean value of ecological variables associated with clusters increased or decreased with the transition event. The decreasing label in the panel legend means that the cluster transition occurred within a negative ecological gradient in which the values of the variables included in the category (Host, Land, or Meteo) decrease (and vice-versa for the increasing label). In panels (**D and E**), only transitions related to the European 3 clonal complex are presented, for which we implemented phylodynamic inference.

The spatial distribution of the four ecological clusters showed geographical consistency across the study area (Fig. 6A). Together, the first two dimensions of the MFA represented 61.6% of the total variance among the 170 samples (Fig. 6B), revealing inherent correlative data structures. For example, while there was a positive correlation between the densities of red deer and mufflon, and independently between wild boar and bovine, these two groups of animals were negatively correlated. We mapped each sample and corresponding ecological cluster to the two first dimensions of the MFA (Fig. 6C), which further revealed inherent data structures of interest (Figure S8). Ecological cluster I (*n* = 94) was characterized by a combination of high mufflon and red deer densities together with the highest mean annual temperature (Fig. 6BC). On the other hand, ecological cluster II (*n* = 11) was associated with high agriculture and low forest land coverage, high bovine density and road density, and low mean annual temperature. Ecological cluster III (*n* = 35) was not represented among the Eu3 samples (Fig. 6A), but among the remaining samples, it appeared as an outlier of ecological cluster I with a much lower mean annual temperature (Figure S8). Finally, ecological cluster IV (*n* = 30) was linked to intermediate levels of several ecological variables and particularly low density of fallow deer and bovine. A full visual description of the ecological gradients across samples and clusters is in Figure S8.

Regarding Eu3 samples alone, phylogeographic transition probabilities between and within ecological clusters were estimated. The symmetric assumption showed high support for transitions between ecological clusters I and IV (PP = 0.89) and between ecological clusters II and IV (PP = 1.00). This was consistent with the inferred transition probabilities allowing asymmetry, where transitions from ecological cluster I to IV (PP = 0.84), from II to I (PP = 0.66), and from II to IV (PP = 1.00) had high support. In general, the phylogeographic inferred events between ecological clusters were dominated by within-cluster events, especially within clusters I and II. Similar to inferences related to diversity, *N*_e_, and municipality transitions (Fig. 3B, C and 5A), the turn of the century was characterized by an increase in the frequency of transitions within clusters I and II (Fig. 6D). Among the between-cluster transitions, those from II into IV were the most frequent, consistent with the highest inferred probability of cluster transitions and likely related to their spatial proximity (Fig. 6A). We also summarized between-cluster transition events according to whether the cluster mean value of ecological variables increased or decreased with the transition event (Fig. 6E). Most transitions took place on a positive gradient among the *Meteo* category (to higher temperature and precipitation), and negative gradients for both the *Land* (to lower agriculture and road density) and *Host* (to lower host densities) categories (Fig. 6E).

## DISCUSSION

This study reports an eco-phylodynamic approach to *M. bovis* within the hotspot risk area of Portugal, representing a multi-host transmission system at the livestock-wildlife interface within a rich ecological landscape. We focused our analyses on a single, newly identified clade related to the Eu3 clonal complex. This is the first study focusing on Eu3 in Iberia, which lacks epidemiological and evolutionary understanding.

Regarding the entire data set, the SNP-based maximum likelihood tree discriminated four major clades, corresponding to four different clonal complexes of which Eu2 (73%) and Eu3 (25%) were the most representative. This was in agreement with previous studies reporting the prevalence of clonal complexes in Portugal (16, 19, 36). The SNP distance analysis highlighted local networks, with the geographic distribution of SNP clades suggesting the natural circulation of particular subclades for long periods of time. The observation of several distantly related lineages present in the same region further suggested the occurrence of long-time past introduction events, whose origin could not be determined but is likely to be from regions across the Spanish border. Alternatively, past spatial segregation of TB subpopulations could have led to similar population structures as the ones observed in this study. Disease maintenance was evident in hunting areas since transmission events occurred between different wildlife hosts for long periods of time, with 2008–2016 being the longest period recovered. In previous studies using gold-standard molecular methods, such as spoligotyping and MIRU-VNTR, TB emergence and persistence in wildlife populations have been shown (16). Disease persistence in wildlife may result from the passive control strategies that have been applied in Portugal (11) and from the environmental persistence of *M. bovis* in animal aggregation points, such as drinking and feeding spots (37). National authorities tackle animal TB mainly through culling programs focused on cattle, overlooking transmission in wildlife, control of wild ungulates overpopulation, and infection via the environment.

Temporal signal analysis was performed as a prerequisite to explore *M. bovis* evolutionary history in more detail. A weak temporal signal was present when considering the entire genome data set, which is commonly reported in studies concerning the *M. tuberculosis* complex (38, 39), even when exploring much broader data sets, reflecting the difficulties of studying slowly- and clonally-evolving microorganisms. We, thus, focused on the Eu3 clonal complex, showing clock-like behavior (even if moderate), which represented a quarter of the samples from the animal TB hotspot risk area of mainland Portugal.

We were able to infer *M. bovis* MRCA of the Eu3 clonal complex to have existed around three decades ago, with a substitution rate similar to previous studies regarding Eu2 clonal complex (40–42), but lower than the one recently reported for the Eu3 clonal complex (0.42–0.57 substitution/genome/year) (30). The MRCA was inferred to have been associated with bovine, with subsequent host transition events towards wildlife species, with some reverse host transition from wildlife reservoirs back to bovine also occurring later. There was, thus, support for both intra- and inter-species transmission routes between all host species, reinforcing the occurrence of historical and sustained cross-species transmission within this ecosystem (5, 6, 16, 19). The phylogeographic inference also revealed that the MRCA was associated with the Portalegre district (probably from Castelo de Vide municipality) with subsequent geographic transition events towards the Castelo Branco district, and reverse host transition from Castelo Branco back to Portalegre also occurring more recently. Although not possible to demonstrate with the current data set, this seemingly free geographic transmission range may likely occur due to animal movement (namely wildlife), bovine trading, or both, which are known risk factors for animal TB spread (43–45).

Phylogeographic inferences using ecological clusters revealed that transmission events between different ecological settings were dominated by events between two clusters: clusters II and IV, located in Portalegre and on the border between Portalegre and Castelo Branco, respectively. There was high support for both symmetric and asymmetric probabilities between the clusters. However, assuming asymmetric probabilities resulted in most events being estimated from cluster II into IV. Cluster II was the most involved in all inferred cluster transmission events adding to 48% of the total, being also part of 79% of between-cluster events. While our analysis remained purely qualitative without the capacity to infer causation, its outputs were compatible with the notion that wild animals foraging into or away from cluster II, which is dominated by areas of higher bovine, agriculture, and road densities, could play a role in TB dissemination in this hotspot area. Indeed, between-cluster events were associated with measurable ecological gradients: a positive gradient among climate-related variables towards higher temperature and precipitation, a negative “humanized” gradient (towards higher forest, lower agriculture, lower road density), and a negative gradient of host densities (from high to low density among most hosts). Altogether, these results stress that TB is an established wildlife disease without strict geographic or ecological barriers.

Given that bovine populations are far more restricted in movement than wildlife populations, and in light of the inferred transmission events sourced at ecological cluster II in Portalegre towards cluster IV bordering and including Castelo Branco, these results suggest that existing TB control measures are insufficient to prevent long-distance transmission and spillover within the livestock-wildlife interface. These observations unravel the bordering region between the two districts, within the much larger officially declared hotspot risk area, as a potential corridor of unrecognizsed importance for the maintenance of *M. bovis*. Indeed, they support the importance of wildlife species in *M. bovis* dissemination in the Portuguese hotspot risk area and the need for new interventions targeting livestock-wildlife interactions.

For example, the population expansion of both wild boar and red deer in Europe, including in the Iberian Peninsula (46–49), is a known risk factor for disease expansion due to animal dispersion, higher animal densities, and aggregation, together with the sympatric character of both species. New strategies focusing on these wildlife reservoirs should be considered to substantially improve the control of animal TB in Portugal. In this area, investment in upscaling sampling and WGS efforts will be critical, as it is the main way to improve data-driven reconstruction of local spatio-temporal histories of *M. bovi*s, essential to design such control strategies.

Both Eu2 and Eu3 *M. bovis* clonal complexes exhibited comparable substitution rates (0.3 in Eu2 vs 0.2 in Eu3), yet the estimation for the MRCA of Eu2 extended further back in time (1777) compared to Eu3 (1991) (35). This finding reflects the historical spread of Eu2 isolates in Portugal, as well as in other regions historically linked to our country (e.g., Brazil) (28, 50), and the more recent introduction of the Eu3 clonal complex into the national territory.

Regarding host transmission analyses, genomic data for the Eu3 clonal complex suggested a cattle origin for the MRCA, while the Eu2 clonal complex MRCA was linked to red deer (35). Moreover, within the Eu3 clonal complex, high transition rates indicated transmission across all host species. However, in the Eu2 clonal complex data set, transmission between cattle and red deer was less probable (35). These epidemiological distinctions between *M. bovis* clonal complexes may stem from differences in host ecology, host tropism, or different host excretion patterns. Specifically, wild boar exhibits a broader generalist behavioral spectrum, as it forages across agroforestry and pasture regions shared with cattle, fostering interaction (9, 10, 51, 52). Additionally, wild boar coexists with red deer, which typically inhabit forested areas, with lower anthropic perturbance, thereby wild boar might connect livestock and wild communities individuals from both ecological niches (9, 10, 51, 52). Moreover, red deer tend to avoid direct contact with other species, while wild boar and cattle tolerate other species presence.

In sum, the Iberian Peninsula as a whole requires new, improved, and innovative methods to inform science-based management decisions in the ecological interfaces between livestock and wildlife to prevent the overflow of TB and other epidemics.

So far, the implemented measures appear insufficient to prevent between-species transmission across the wildlife-livestock interface. Thus, an improvement of management actions towards reducing contacts and mixing in this interface is critical if aiming to achieve prevalence levels that may sustain strict control or even eradication. This is particularly relevant at first instance for livestock, as it remains the most accessible and manageable host population within which additional control measures may lead towards eradication. Together, increased surveillance of livestock areas adjacent to wildlife hotspot areas or highly dense areas needs to occur due to the detected high transition rates within cattle. Moreover, clear measures to reduce and monitor both livestock and wildlife movements, which seem to be highly responsible for transmission, need to be rigorously taken, particularly in extensive livestock-producing areas and managed hunting areas. Finally, a strong investment effort is needed to find answers to critical questions on which effective control measures depend upon, namely further studies need to be conducted to clarify: the role of other species as intermediate hosts; animal movements and contact patterns as important epidemiological links; and ecological wildlife parameters, such as animal density; and environmental parameters, such as bioclimatic variables, that may influence transmission and TB spread. Our study contributes to this fragmented landscape of control and surveillance by identifying for the first time the bordering region between Portalegre and Castelo Branco as playing a central role in local and long-range dissemination, and perhaps extra priority efforts should first be focused on this region within the much larger official hotspot risk area of Portugal.

## MATERIALS AND METHODS

### Sampling

*M. bovis* isolates (*n* = 170) were sampled from bovine (*n* = 51), red deer (*n* = 66), and wild boar (*n* = 53) from the hotspot risk area of mainland Portugal [districts: Castelo Branco (*n* = 117), Portalegre (*n* = 53)] bordering Spain (Table S1). Sampling was performed under an official context according to national legislation (see reference (19) for details). Specifically, animal samples were collected in the scope of the national control and eradication bovine TB program, which is managed by the national veterinary authority, and processed for culture according to standard OIE guidelines. European 1 and 2 genomes were previously published (19, 35), with sequence reads obtained from the National Centre for Biotechnology Information SRA database, deposited under Bioproject accession numbers PRJNA682618 and PRJNA946560. The remaining sequences were generated *de novo*.

### DNA extraction

Novel *M. bovis* isolates were stored at −80°C in the National Reference Laboratory of Animal Tuberculosis (INIAV, IP), re-cultured, and genomic DNA extracted as in reference (19).

### Sequence Curation

Genomic DNA was commercially sequenced (Eurofins, Konstanz, Germany) using the Illumina NovaSeq platform (paired-end 150 bp) according to the manufacturer’s specifications. Raw read FASTQ files of all 170 isolates were used for quality control evaluation, trimmed, filtered, and taxonomically classified as in reference (35). Samples were analyzed using the vSNP pipeline available at https://github.com/USDA-VS/vSNP (accessed: 1 June 2021) as in reference (19). The average depth and genome coverage were 272.7× and 99.69% (respectively). The SNP alignment had a total of 4,092 polymorphic positions (Table S2).

### Transmission mapping

The SeqTrack R library (53, 54) was used to infer local transmission networks through genomic distance minimization between isolates and to keep sampling dates coherent. Transmission trees were constructed with cases grouped within the same transmission chain using a cut-off value of five SNPs within a time frame of 5 years (55, 56). Choosing an optimal cut-off can be somewhat subjective, which has been shown by the variety of SNP cut-offs (5–20) that have been used to identify clusters of potentially linked TB cases (32). Also, this threshold may vary within the epidemiology of different *M. bovis* lineages/clonal complexes.

### Phylogenomic analyses

A maximum likelihood method defined with GTR gamma-distributed with invariant sites and four discrete gamma categories, including 1,000 bootstrap inferences, was implemented in MEGA-X v10.1.8 (57). Lineage identification was carried out using KvarQ version 0.12.2 (58) for the assessment of lineage-specific SNPs as in reference (34).

Temporal signals were measured with TempEst v1.5.3 (59) and LSD v0.3-beta (60). The best-fitting nucleotide substitution model was selected by Bayesian information criteria (BIC) implemented in jModeltest2 v2.1.10 (61). Several Bayesian coalescent MCMC analyses were performed in BEAST2 v2.6.2 (62) using the best-fitting nucleotide substitution model together with three molecular clock models and three coalescent demographic priors, resulting in nine different models.

A DATM analysis (63) was implemented in BEAST2 using host species as a discrete trait (bovine, red deer, and wild boar), for both symmetric and asymmetric analysis using the best fitting model (TIM2 with relaxed exponential clock and Bayesian skyline population).

Full details are in Text S1.

### Ecological clustering

With reference to sample geographical location, 12 ecological variables were sourced from publicly available databases and associated with each sample. We used a heuristic approach based on classic ecology methods (Ward’s clustering, fusion levels) to define groups of samples sharing similar background ecological environments (here termed ecological clusters). To intuitively explore the relationship of ecological clusters with identified lineages and transmission events, we performed multiple factor analysis (MFA, based on principal component analysis) by grouping variables into three categories: *host*, *land*, and *meteo* (i.e., related to host-species, landscape, and climate, respectively).

Full details on variables used and a step-by-step description of the heuristic clustering and MFA are in Text S2.

### Phylogeographic analyses

We performed both geographic and ecological phylodynamic analyses. For geographic analyses, we separately used discrete (sample district or municipality) and continuous (sample latitude-longitude) variables. For ecological analyses, we used the assigned sample cluster as a discrete variable (see Ecological clustering). Phylogeographic inferences used a coalescent DATM with both symmetric and asymmetric assumptions with the best fitting model (see Phylogenomic analyses).

Full details are in Text S1.

## Data Availability

The sequence data included in this work are deposited under Bioproject accession numbers PRJNA682618 and PRJNA946560 at a public domain server in the National Centre for Biotechnology Information (NCBI) SRA database. All the tables describing the samples and their metadata are provided as supplemental Excel files.

## SUPPLEMENTAL MATERIAL

Supplemental material spectrum.03829-23-s0001.docx Supplemental text, tables, and figures.

Supplemental tables spectrum.03829-23-s0002.xlsx Tables S1, S2, and S4.

ASM does not own the copyrights to Supplemental Material that may be linked to, or accessed through, an article. The authors have granted ASM a non-exclusive, world-wide license to publish the Supplemental Material files. Please contact the corresponding author directly for reuse.
